# Application of multimodal ultrasound in the biomechanical evaluation of carotid intima-media thickness in type 2 diabetes mellitus: a focus on subclinical vascular changes

**DOI:** 10.3389/fendo.2026.1758096

**Published:** 2026-04-15

**Authors:** Xuhong Nan, Linmei Tang, Xue Fang, Xinlei Yang, Yu Kang

**Affiliations:** 1 School of Medicine and Life Sciences, Chengdu University of Traditional Chinese Medicine, Chengdu, China; 2 Department of Ultrasound Medicine, Hospital of Chengdu University of Traditional Chinese Medicine, Chengdu, China; 3 Department of Ultrasound Medicine, The First People’s Hospital of Longquanyi District, Chengdu, China

**Keywords:** carotid intima-media thickness, hemodynamics, pulse wave velocity, type 2 diabetes mellitus, wall shear stress

## Abstract

**Objective:**

To assess multimodal ultrasound technology in the biomechanical assessment of carotid intima-media thickness (cIMT) in patients with type 2 diabetes mellitus (T2DM), specifically focusing on those with increased cIMT but without atherosclerotic plaques or significant stenosis.

**Methods:**

In this exploratory cross-sectional study, 65 T2DM patients (thickened vs. normal cIMT) and 27 controls were examined using color Doppler ultrasound flow imaging (CDFI), ultrasound vector flow imaging (V-Flow), and ultra-fast pulse wave velocity (UF-PWV). Measurements included peak systolic velocity (PSV), end-diastolic velocity (EDV), pulsatility index (PI), resistance index (RI), wall shear stress (WSS), pulse wave velocity at the beginning of systole (PWV-BS), and pulse wave velocity at the end of systole (PWV-ES). Differences across groups and independent factors associated with cIMT were analyzed via correlation and multivariable linear regression.

**Results:**

The T2DM group exhibited higher HbA1c levels than the control group (*P* < 0.001). The T2DM with thickened cIMT group exhibited significantly lower EDV (*P* = 0.005) and higher Body Mass Index (BMI) (*P* = 0.006) than the control group. Furthermore, the same group showed significantly lower mean WSS (WSSmean) than the control and T2DM with normal cIMT groups (*P* < 0.05). The T2DM with thickened and normal cIMT groups showed significantly higher PWV-ES than the control group (*P* < 0.05). A positive correlation was observed between cIMT and BMI (*ρ* = 0.392, *P* < 0.001), HbA1c (*ρ* = 0.425, *P* < 0.001), and PWV-ES (*ρ* = 0.506, *P* < 0.001). Both WSSmean (*ρ* = −0.365, *P <* 0.001) and EDV (*ρ* = −0.209, *P* < 0.05) were negatively correlated with cIMT. After adjustment for clinical covariates, BMI, WSSmean, and PWV-ES were identified as independent factors associated with cIMT in the overall cohort. In the T2DM group, PWV-ES (*B* = 0.034, *P* = 0.004) and WSSmean (*B* = −0.060, *P* = 0.039) remained independently associated with cIMT.

**Conclusion:**

Preliminary findings suggest V-Flow and UF-PWV imaging may detect biomechanical alterations in T2DM patients. While these techniques may offer insights into subclinical vascular remodeling prior to plaque formation, this cross-sectional study should be cautiously interpreted as hypothesis-generating.

## Introduction

Atherosclerosis (AS) is a major cause of cardiovascular and cerebrovascular events. Its development and progression are closely associated with endothelial dysfunction, changes in blood flow dynamics, and arterial structural remodeling. Abnormal blood flow patterns can directly harm vascular endothelial cells through mechanical stress, resulting in inflammatory responses and lipid accumulation. While carotid atherosclerosis (CA) represents a continuum of disease severity, ranging from increased carotid intima-media thickness (cIMT ≥ 1.0 mm) to carotid plaque and luminal stenosis (1, 2), this study focuses specifically on the initial phase of this spectrum: subclinical cIMT remodeling. Globally, the prevalence of increased cIMT in the population aged 30–79 reached 27.6% in 2020, with the Western Pacific region carrying the highest disease burden (3). In recent years, new assessment metrics based on biomechanical principles have provided notable advantages. One such metric is wall shear stress (WSS), which measures the shear stress exerted by blood flow on the vascular endothelium. Abnormally low WSS levels have been demonstrated to accelerate lipid accumulation and plaque formation owing to mechanisms related to endothelial dysfunction (4, 5). Another important metric is pulse wave velocity (PWV), which measures vascular elasticity by quantifying arterial stiffness. Elevated PWV levels are strongly associated with an increased risk of adverse cardiovascular events (6, 7). Diabetes is a major risk factor for the initiation and progression of AS. Notably, epidemiological evidence indicates that the association between diabetes and increased cIMT is substantially stronger than its association with plaque formation (3, 8). This suggests that increased cIMT serves as a more sensitive subclinical marker for identifying early vascular injury in the diabetic population, providing a critical window for primary prevention before irreversible structural damage occurs (9). By focusing on increased cIMT in the absence of overt plaque, we can better isolate and evaluate the initial biomechanical shifts that characterize early-stage vascular remodeling, which may otherwise be confounded by the complex flow patterns associated with advanced lesions. Therefore, this study aims to utilize various ultrasound techniques to exploratively detect changes in hemodynamics and vascular wall function during the stages of isolated increased cIMT in individuals with type 2 diabetes mellitus (T2DM), without the presence of plaque or significant stenosis.

## Methods

### Study population

This study enrolled patients diagnosed with T2DM who visited the Endocrinology Department at the Affiliated Hospital of Chengdu University of Traditional Chinese Medicine between November 2023 and April 2024. All diagnoses were made according to the criteria outlined in the 2020 Chinese Guidelines for the Prevention and Treatment of Type 2 Diabetes (10). The exclusion criteria were ① carotid artery plaque or carotid stenosis with diameter reduction ≥ 50%, ② left ventricular ejection fraction < 55%, ③ heart valve disease, ④ long-term smoking history, ⑤ intracranial (specifically ischemic or hemorrhagic stroke and TIA) or peripheral vascular disease, ⑥ uncontrolled hypertension (BP > 140/90 mmHg), ⑦ severe hepatic or renal insufficiency, ⑧ age < 30 years, and ⑨ cardiac arrhythmias (e.g., atrial fibrillation, other significant irregular rhythms). Ultimately, 65 patients were included and divided into two groups based on the intima-media thickness (IMT) of the distal left common carotid artery (predefined as the measurement side for all participants): normal cIMT (cIMT < 1.0 mm) and thickened cIMT (1.0 ≤ cIMT < 1.5 mm). Concurrently, 27 non-diabetic subjects recruited from the Department of Endocrinology (primarily for obesity or thyroid-related consultations) were enrolled as the control group. This group had no history of diabetes and met all the aforementioned exclusion criteria.

### Instruments and measurement

#### Instruments

Examinations were conducted using the Mindray Resona R9 ultrasound diagnostic system (Shenzhen Mindray Bio-Medical Electronics Co., Ltd.), equipped with an L9–3 linear array probe (3.0–9.0 MHz). All ultrasound examinations were performed on the same system by a single physician who had received standardized training in vascular ultrasound to ensure measurement consistency. The system features a built-in automatic measurement technology for cIMT, vector flow imaging (V-Flow) and ultra-fast pulse wave velocity imaging (UF-PWV) technologies, and quantitative analysis software.

#### The cIMT measurement

**Figure 1:**
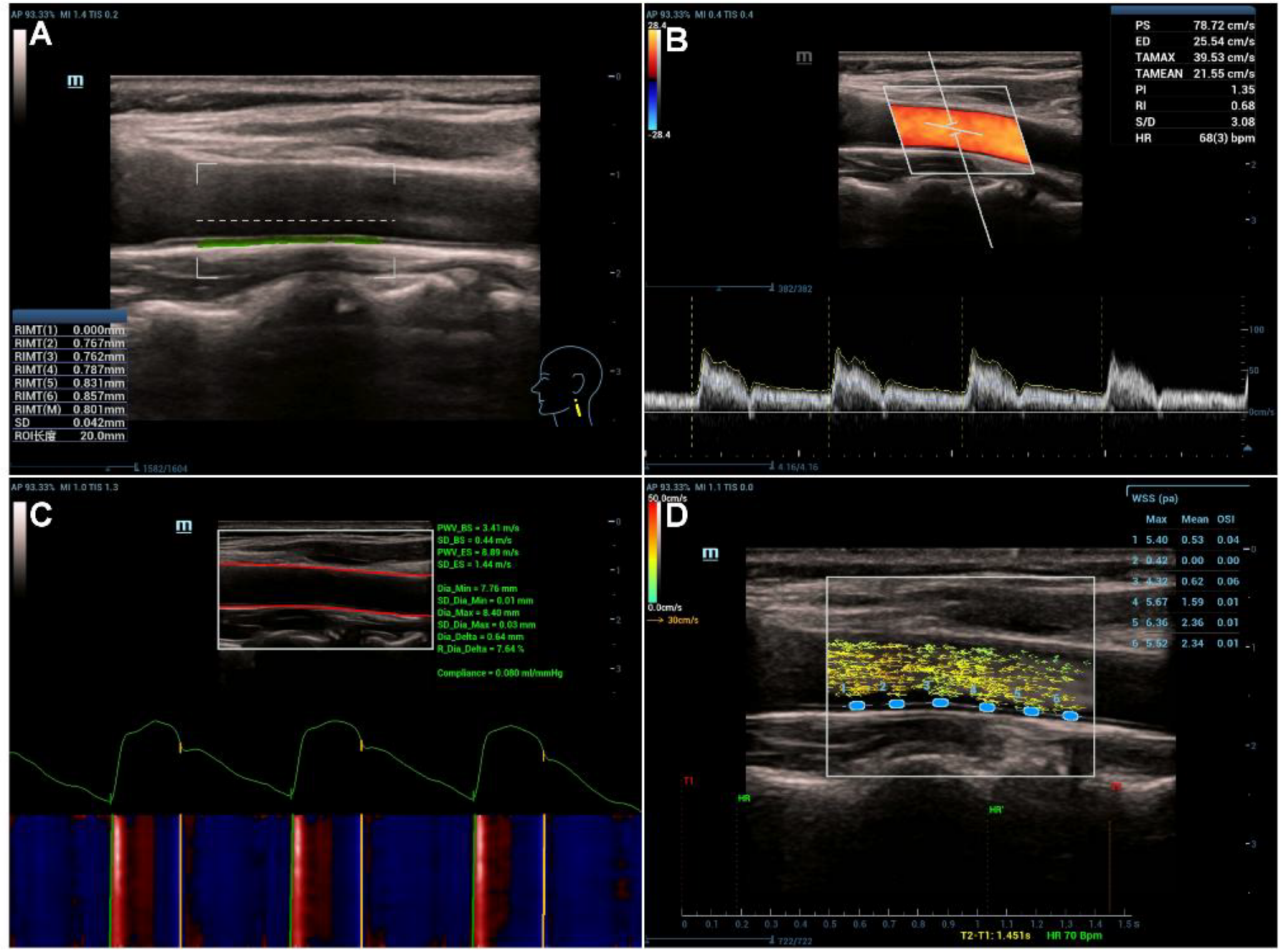
Representative images of carotid vascular assessment using high-frame-rate ultrasound. **(A)** Automated measurement of the carotid intima-media thickness (cIMT). **(B)** Hemodynamic assessment of the carotid artery, showing color Doppler flow imaging (top) and the pulsed-wave Doppler spectrum (bottom) for the measurement of peak systolic velocity (PSV) and end-diastolic velocity (EDV). **(C)** Assessment of arterial stiffness using pulse wave velocity (PWV) technology, showing PWV at the beginning (PWV-BS) and end (PWV-ES) of systole. **(D)** Vector Flow (V-Flow) mapping for the visualization of complex flow patterns and the quantification of wall shear stress (WSS) within a 20 mm vessel segment.

The patients were examined in the supine position, with their necks relaxed and heads slightly turned to the opposite side. The distal common carotid artery was visualized in a longitudinal view in accordance with the American Society of Echocardiography (ASE) guidelines (11). The ultrasound beam was oriented perpendicular to the anterior and posterior walls of the vessel using the carotid imaging mode. Then, the automatic cIMT measurement software was activated, and a region of interest was designated on the posterior wall of the common carotid artery. Six consecutive automatic measurements were obtained, and the mean value was calculated (**Figure 1A**).

#### Hemodynamic parameters measurement

The distal common carotid artery was examined via color Doppler flow imaging (CDFI). The pulse repetition frequency and baseline were adjusted to display the systolic velocity waveform without aliasing, with the peak systolic velocity occupying approximately two-thirds of the spectral display. A low wall filter (50–100 Hz) was applied during acquisition. Pulsed-wave Doppler was activated, with the sample volume set to approximately one-third of the lumen diameter and positioned at the center of the vessel. The angle between the Doppler beam and the direction of blood flow was maintained at < 60°. Spectral envelopes from three consecutive cardiac cycles were automatically traced by the system, with manual correction performed when necessary. The peak systolic velocity (PSV), end-diastolic velocity (EDV), pulsatility index (PI), and resistance index (RI) of the distal common carotid artery were measured (**Figure 1B**). All examinations were performed after a resting period of 5–10 minutes. Participants remained calm and breathed spontaneously during data acquisition. Measurements were averaged over three cardiac cycles, and individuals with arrhythmias were excluded.

#### PWV measurement

The distal segment of the common carotid artery was visualized, and the vascular pulse wave was analyzed. The system automatically measured the pulse wave velocity at the beginning and end of systole (PWV-BS and PWV-ES) (**Figure 1C**). Measurements were performed unilaterally on the same predefined side. Each parameter was acquired three to five times, and the median value was used for subsequent analysis.

#### WSS measurement

The distal common carotid artery was examined, and the vector flow imaging (V-Flow) mode was activated to automatically obtain vector flow images. Six measurement points were evenly placed along the posterior wall of the artery within a 20 mm vessel segment. Wall shear stress was measured at each point throughout the cardiac cycle, and the time-averaged wall shear stress (TAWSS) across the six points was calculated. The number of measurement points was set according to the maximum supported by the system (**Figure 1D**).

### Data collection

Demographic and clinical data, including age and sex, were retrospectively retrieved from the hospital’s electronic medical records. Blood pressure values were recorded from the initial physical examination upon admission. Laboratory parameters, including lipid profile (total cholesterol, triglycerides, HDL-C, LDL-C) and glycated hemoglobin (HbA1c), were obtained from routine fasting venous blood tests analyzed by the hospital’s central laboratory. To address potential confounding factors related to pharmacological treatment and disease progression, patient medication history – specifically detailing current antidiabetic therapy, lipid-lowering therapy, and antiplatelet therapy – was additionally collected. Furthermore, the diabetes duration was systematically extracted. This supplementary clinical data was primarily sourced from electronic medical records and, where details required clarification or were incomplete, obtained through structured telephone interviews with patients. All clinical and laboratory data included in the analysis were collected during the same hospitalization as the ultrasound examination to ensure temporal consistency.

To minimize bias, all ultrasound examinations were performed by a senior sonographer blinded to the subjects’ clinical history and laboratory results. Furthermore, clinical data collection and ultrasound parameter extraction were conducted by independent researchers to ensure the objectivity of the findings. Measurement reproducibility was assessed in 30 randomly selected participants. Intra-observer reliability was determined by the same sonographer re-analyzing images after a two-week interval, while inter-observer reliability was evaluated by a second senior sonographer. Agreement was quantified using intraclass correlation coefficients (ICCs).

### Statistical methods

Data analyses were performed using SPSS version 26.0 (IBM Corp., NY, USA). Continuous variables with normal distribution were expressed as mean ± standard deviation, while non-normally distributed variables were presented as median and interquartile range [*M* (*P*25, *P*75)]. Categorical data were expressed as counts (percentages) and compared using the chi-square test. Comparisons among the three groups were conducted using one-way analysis of variance (ANOVA) for normally distributed data, and the Kruskal-Wallis H test for non-normally distributed data. Bonferroni correction was applied for *post hoc* pairwise comparisons. Correlations between cIMT and other continuous variables were assessed using Spearman’s rank correlation analysis. To identify independent factors associated with cIMT, linear regression analyses were performed. First, univariate linear regression analyses were conducted to examine the associations between clinical, biochemical, and hemodynamic variables and cIMT. Variables with a *P* value < 0.20 in the univariate analysis were included in the multivariable linear regression model. Multivariable analyses were performed in the entire cohort and separately in the diabetic subgroup. In the diabetic subgroup analysis, diabetes duration, antidiabetic therapy, antiplatelet therapy, and lipid-lowering therapy were additionally forced into the model as clinically relevant covariates. Inter- and intra-observer reliability for the ultrasound parameters were evaluated using the intraclass correlation coefficient (ICC) with 95% confidence intervals (CIs). ICC values > 0.75 were considered to indicate excellent reproducibility. A two-sided *P* < 0.05 was considered statistically significant.

## Results

### General characteristics

Initially, 95 participants were screened. Three subjects aged < 30 were excluded according to the exclusion criteria. Consequently, 92 participants were enrolled, comprising 27 controls and 65 patients with T2DM. The T2DM group was further categorized into a normal cIMT subgroup (*n =* 30) and a thickened cIMT subgroup (*n =* 35). The T2DM group had significantly higher HbA1c levels than the control group (*P* < 0.001). Furthermore, the thickened cIMT group exhibited a significantly higher BMI compared to the control group (*P* = 0.006). No statistically significant differences were observed among the three groups in terms of age, blood pressure, sex or lipid profiles (*P* > 0.05). Within the T2DM group, diabetes duration was comparable between the normal cIMT and thickened cIMT subgroups (P > 0.05). Similarly, the proportion of patients receiving antidiabetic and lipid-lowering therapies did not differ significantly between the normal cIMT and thickened cIMT subgroups (all *P* > 0.05). Notably, all five insulin-treated patients in our T2DM cohort were exclusively found within the thickened cIMT subgroup. Moreover, antiplatelet therapy was used by 11.8% (4/34) of the thickened cIMT group, with no users in the normal cIMT or control groups, as shown in **Table 1**.

**Table 1:** Comparison of baseline characteristics among the control, T2DM with normal cIMT group, and T2DM with thickened cIMT group.

Characteristics	Control group (*n =* 27)	T2DM		*P* value
		Normal cIMT group (*n =* 30)	Thickened cIMT group (*n =* 35)	
Age (years)	50.3 ± 7.3	52.6 ± 6.1	54.0 ± 5.7	0.092
Male n (%)	14 (51.9%)	15 (50.0%)	18 (51.4%)	0.989
BMI (kg/m²)	22.7 (22.2, 23.5)	23.6 (22.9, 23.9)	23.8 (23.1, 24.2) ^**^	0.001
HbA1c (%)	4.93 ± 0.51	9.37 ± 1.80 ^***^	10.07 ± 2.53 ^***^	<0.001
HDL (mmol/L)	1.44 ± 0.10	1.29 ± 0.13	1.25 ± 0.16	0.057
LDL (mmol/L)	2.87 ± 0.26	2.95 ± 0.19	2.96 ± 0.24	0.221
TG (mmol/L)	1.23 ± 0.30	1.32 ± 0.42	1.41 ± 0.37	0.657
TC (mmol/L)	4.93 ± 0.33	4.81 ± 0.27	4.82 ± 0.39	0.302
SBP (mmHg)	116.7 ± 7.1	119.5 ± 7.3	122.0 ± 9.3	0.058
DBP (mmHg)	73.6 ± 5.2	75.4 ± 5.2	76.4 ± 6.8	0.081
Diabetes duration (years)	–	2 (1.0, 6.8)	4 (2.9, 7.0)	0.129
Antidiabetic treatment	–	24/27 (88.9%)	33/34 (97.1%)	0.313
Lipid-lowering treatment	–	6/27 (22.2%)	11/34 (32.4%)	0.408
Antiplatelet therapy	–	0/27 (0.0%)	4/34 (11.8%)	0.122

BMI, Body Mass Index, HbA1c: Glycated hemoglobin, HDL: High-density lipoprotein, LDL: Low-density lipoprotein, TG: Triglycerides, TC: Total cholesterol, SBP: Systolic Blood Pressure, DBP: Diastolic Blood Pressure.

Percentages for drug history were calculated based on the number of patients with available medical records.

*Post hoc* Bonferroni test: ^**^P < 0.01 vs control group, ^***^P < 0.001 vs control group.

### Comparison of carotid biomechanical parameters

The T2DM with thickened cIMT group had significantly lower EDV than the control group (*P* = 0.005). No statistically significant differences were observed among the three groups in terms of PSV, PI, or RI (*P* > 0.05). The thickened cIMT group exhibited significantly lower WSSmean than the control and T2DM with normal cIMT groups (*P* < 0.05). No significant differences were observed between the control and normal cIMT groups in terms of WSSmean (*P* > 0.05). PWV-ES exhibited a progressive increase from the control group to the normal cIMT group and further to the thickened cIMT group (*P* < 0.05). Contrarily, no significant differences were observed in PWV-BS among the three groups (*P* > 0.05), as shown in **Table 2**.

**Table 2:** Comparison of ultrasound parameters among the control, T2DM with normal cIMT, and T2DM with thickened cIMT groups.

Parameters	Control group (*n =* 27)	T2DM		*P* Value
		Normal cIMT group (*n =* 30)	Thickened cIMT group (*n =* 35)	
PSV (m/s)	0.96 ± 0.17	0.89 ± 0.18	0.86 ± 0.18	0.111
EDV (m/s)	0.30 ± 0.06	0.28 ± 0.04	0.26 ± 0.05 ^**^	0.005
PI	1.37 ± 0.26	1.38 ± 0.27	1.46 ± 0.28	0.302
RI	0.64 ± 0.06	0.67 ± 0.06	0.68 ± 0.07	0.971
PWV-BS (m/s)	4.90 ± 0.91	5.32 ± 1.61	5.40 ± 2.17	0.496
PWV-ES (m/s)	6.87 (2.80)	8.66 (1.93) ^*^	9.31 (3.16) ^***^	0.001
WSSmean (Pa)	2.32 ± 0.71	2.07 ± 0.82	1.52 ± 0.86 ^***†^	0.001

PSV, peak systolic velocity, EDV, end-diastolic velocity, PI, pulsatility index, RI, resistance index, PWV-BS, pulse wave velocity at the beginning of systole, PWV-ES, pulse wave velocity at the end of systole, WSSmean: mean wall shear stress, cIMT, carotid intima-media thickness, T2DM, type 2 diabetes mellitus.

*Post hoc* Bonferroni test: ^*^P < 0.05 vs control group, ^**^P < 0.01 vs control group, ^***^P < 0.001 vs control group, ^†^P < 0.05 vs T2DM with normal cIMT group.

### Reliability analysis

The reliability analysis demonstrated excellent intra- and inter-observer consistency for all evaluated vascular parameters (*P* < 0.001), as shown in **Table 3**.

**Table 3:** Intra- and inter-observer reliability of the carotid vascular parameters.

Parameters	Intra-observer reliability		*P*-value	Inter-observer reliability		*P*-value
	ICC	95% CI		ICC	95% CI	
WSSmean	0.98	(0.85, 0.99)	< 0.001	0.98	(0.97, 0.99)	< 0.001
cIMT	0.88	(0.74, 0.95)	< 0.001	0.96	(0.92, 0.98)	< 0.001
EDV	0.98	(0.97, 0.99)	< 0.001	0.83	(0.63, 0.93)	< 0.001
PWV-ES	0.97	(0.91, 0.99)	< 0.001	0.97	(0.93, 0.99)	< 0.001

WSSmean, mean wall shear stress, cIMT, carotid intima-media thickness, EDV, end-diastolic velocity, PWV-ES, pulse wave velocity at the end of systole.

### Correlation analysis

Carotid IMT exhibited significant positive correlations with BMI (*ρ* = 0.392, *P* < 0.001), HbA1c (*ρ* = 0.425, *P* < 0.001), and PWV-ES (*ρ* = 0.506, *P* < 0.001). Conversely, significant negative correlations were observed with EDV (*ρ* = −0.209, *P <* 0.05) and WSSmean (*ρ* = −0.365, *P* < 0.001), as shown in **Table 4**.

**Table 4:** Correlation analysis of carotid intima-media thickness with clinical and vascular parameters.

Variables	*ρ*	*P* value
Age	0.116	0.272
BMI	0.392	< 0.001
HbA1c	0.425	< 0.001
LDL	0.145	0.169
HDL	-0.062	0.560
TG	0.024	0.819
TC	0.198	0.060
WSSmean	-0.365	< 0.001
PWV-ES	0.506	< 0.001
PWV-BS	0.201	0.055
PSV	-0.149	0.157
EDV	-0.209	0.045
PI	0.022	0.835
RI	-0.024	0.822

BMI, Body Mass Index; HbA1c, Glycated Hemoglobin; LDL, Low-Density Lipoprotein; HDL, High-Density Lipoprotein; TG, Triglycerides; TC, Total Cholesterol; WSSmean, Mean Wall Shear Stress; PWV-ES, pulse wave velocity at the end of systole; PWV-BS, pulse wave velocity at the beginning of systole; PSV, peak systolic velocity; EDV, end-diastolic velocity; PI, pulsatility index; RI, resistance index.

### Multivariable linear regression analysis of factors associated with cIMT

Multivariable linear regression analyses were performed to identify independent factors associated with cIMT. In the analysis of the entire cohort, BMI, WSSmean, and PWV-ES were independently associated with cIMT (**Supplementary Table 1**). Specifically, BMI (*B* = 0.021, *P* = 0.044) and PWV-ES (*B* = 0.023, *P* = 0.001) showed positive associations with cIMT, whereas WSSmean was negatively associated with cIMT (*B*= −0.044, *P* = 0.015).

Further analysis restricted to the diabetic subgroup demonstrated that WSSmean and PWV-ES remained independently associated with cIMT (**Table 5**). PWV-ES was positively associated with cIMT (*B* = 0.034, 95% CI: 0.011 ~ 0.056, *P* = 0.004), while WSSmean showed a negative association (*B* = −0.060, 95% CI: −0.110 ~ −0.003, *P* = 0.039). HbA1c showed a borderline association with cIMT (*B* = 0.016, 95% CI: 0.000 ~ 0.032, *P* = 0.051). Other variables, including BMI, diabetes duration, and medication therapies, were not independently associated with cIMT in this subgroup.

**Table 5:** Univariate and multivariate linear regression analysis of factors associated with cIMT.

Variables	Univariate regression analysis				Multivariate regression analysis			
	*B*	SE	95% CI	*P* value	*B*	SE	95% CI	*P* value
Age	0.010	0.005	(0.000, 0.021)	0.044	0.006	0.005	(-0.004, 0.016)	0.227
BMI	0.041	0.017	(0.008, 0.074)	0.015	0.021	0.017	(-0.013, 0.055)	0.217
TC	0.039	0.045	(-0.052, 0.130)	0.392				
TG	0.021	0.047	(-0.073, 0.115)	0.653				
HDL	-0.125	0.110	(-0.345, 0.096)	0.262				
LDL	0.051	0.044	(-0.038, 0.139)	0.256				
HbA1c	0.014	0.009	(-0.004, 0.032)	0.140	0.016	0.008	(0.000, 0.032)	0.051
WSSmean	-0.096	0.028	(-0.153, -0.039)	0.001	-0.060	0.027	(-0.110, -0.003)	0.039
PWV-ES	0.048	0.011	(0.027, 0.069)	< 0.001	0.034	0.011	(0.011, 0.056)	0.004
PWV-BS	0.003	0.014	(-0.025, 0.031)	0.837				
EDV	-0.553	0.324	(-1.196, 0.090)	0.091	-0.368	0.510	(-1.393, 0.657)	0.474
PSV	-0.102	0.099	(-0.298, 0.094)	0.305				
PI	0.040	0.064	(-0.087, 0.168)	0.533				
RI	-0.220	0.296	(-0.808, 0.369)	0.460				
Antidiabetic therapy	0.013	0.108	(-0.203, 0.228)	0.907	-0.040	0.096	(-0.232, 0.153)	0.682
Lipid-lowering therapy	0.057	0.059	(-0.061, 0.175)	0.340	0.032	0.055	(-0.078, 0.141)	0.568
Antiplatelet therapy	0.242	0.103	(0.035, 0.448)	0.023	0.065	0.105	(-0.146, 0.276)	0.539
Diabetes duration	0.006	0.008	(-0.009, 0.021)	0.441	0.003	0.007	(-0.012, 0.018)	0.665

cIMT, carotid intima-media thickness.

Values are presented as unstandardized regression coefficients (*B*) with their standard errors (SE) and 95% confidence intervals (CI). Additionally, diabetes duration and pharmacological therapy information were forced into the multivariate model due to their established clinical relevance and potential impact on vascular health.

## Discussion

AS, as the primary pathological basis of ischemic cardiovascular and cerebrovascular diseases, involves a complex interplay of multiple risk factors and underlying mechanisms (12, 13). In the early subclinical stage of this process, increased cIMT serves as a critical surrogate marker, occurring prior to the formation of overt plaques or significant stenosis. Persistent hyperglycemia may impair endothelial nitric oxide bioavailability, thereby contributing to endothelial dysfunction. This dysfunction is associated with increased vascular tension, a procoagulant state, and the release of proinflammatory factors, thereby contributing to early-stage vascular remodeling (14). The unique geometric structure of the carotid artery and its bifurcation render these areas particularly vulnerable to AS. Individual variations, such as excessive bifurcation angles or disproportionate carotid bulb enlargement, can disrupt normal blood flow dynamics (15). Notably, the left carotid artery is documented to be more predisposed to atherosclerotic involvement than the contralateral side (16). Consequently, the left carotid artery was strategically selected as the research site in this study to standardize the measurement environment. This approach aimed to minimize confounding interference from inter-side anatomical and hemodynamic heterogeneity, providing a focused assessment of subclinical changes within the common carotid artery. Research shows that in the pre-plaque stage, before the occurrence of significant morphological changes such as plaque or stenosis, the condition may be associated with metabolic alterations in the extracellular matrix of arterial wall cells, which may affect elastin integrity and vascular biomechanical properties even before overt morphological changes are evident (17). Therefore, the utilization of biomechanical indicators that reflect characteristics such as WSS and vascular elasticity may provide valuable insights for the assessment of cIMT thickening risk.

Conventional color Doppler ultrasound is the most commonly used method for assessment of carotid hemodynamics. The present study found that the T2DM with thickened cIMT group exhibited reduced EDV, which was negatively correlated with cIMT. However, multiple regression analysis revealed that EDV was not an independent factor associated with cIMT. In addition, the measurement of carotid blood flow velocity can be influenced by various clinical conditions (18–20). These limitations suggest that color Doppler ultrasound may be less sensitive for in precise risk assessment during the early subclinical stages of vascular remodeling.

WSS refers to the tangential shear stress exerted on the vascular wall surface during blood flow. In this study, the WSSmean was reduced in the T2DM patients with thickened cIMT group and independently associated with cIMT thickening. Existing research suggests that WSSmean within the physiological range (1.5–2.5 Pa) is a critical mechanical signal for maintaining endothelial homeostasis (21). Conversely, low WSSmean (< 0.5 Pa) may disrupt this protective mechanism, thereby contributing to endothelial dysfunction and lipid deposition under hyperglycemia (22, 23). Therefore, in the context of T2DM, increased cIMT appears to be associated with metabolic abnormalities and reduced WSS. Low WSS promotes lipid and inflammatory cell retention, which may contribute to vascular wall changes (24).

PWV refers to the speed at which pressure waves propagate through the arterial system, reflecting arterial elasticity and stiffness. PWV is slower when arterial elasticity is preserved. When arterial stiffness increases, PWV propagation is accelerated. Abnormally elevated PWV is markedly associated with cardiovascular event risk (25). This study suggests that PWV-ES may serve as a more sensitive biomechanical indicator than PWV-BS and was identified as an independent factor associated with cIMT thickening. Mechanistically, PWV-ES is considered to reflect collagen fiber-dominated structural vascular stiffness. At peak systolic pressure, the arterial wall is fully distended, causing the mechanical load to shift from elastin to the significantly stiffer collagen fibers, which are fully recruited and stretched (26, 27). Its elevation is associated with irreversible pathological remodeling driven by hyperglycemia, including the accumulation of advanced glycation end-products and collagen cross-linking (28). Conversely, PWV-BS primarily reflects smooth muscle-dominated functional elasticity. At the lower pressures of early systole, collagen fibers remain in a folded, non-load-bearing state, allowing the vascular elastic response to be governed primarily by the active tension of smooth muscle cells (29, 30). In the early stages, it may be initially influenced by endothelial dysfunction (e.g., reduced nitric oxide production). However, these changes are easily compensated for and do not directly cause structural alterations (31). As vascular remodeling progresses, structural stiffening may gradually become predominant, potentially allowing PWV-ES to independently identify vascular structural changes in T2DM patients with increased cIMT more sensitively. Current guidelines use a PWV > 10 m/s as a threshold indicating increased arterial stiffness and cardiovascular risk (32).

In addition to hemodynamic parameters, metabolic and clinical factors may influence cIMT (33). In the present study, BMI was identified as an independent factor associated with cIMT in univariate regression analysis but did not retain this association in multivariable analysis, consistent with its role as a cardiovascular risk factor linked to insulin resistance, chronic inflammation, and endothelial dysfunction, which are associated with vascular remodeling and cIMT thickening (34–36). Diabetes duration and pharmacological therapies did not show independent associations with cIMT in the multivariable analysis, although they were included as clinically relevant covariates. Antidiabetic and lipid-lowering agents may contribute to mitigating vascular damage and delaying cIMT thickening in diabetic patients (37–39). One possible explanation is that most patients diagnosed with diabetes in this cohort were already receiving antidiabetic and lipid-lowering therapies. This limited variability may reduce the ability of regression models to distinguish the independent effects of medication on cIMT. Notably, all five insulin-treated patients were exclusively found within the thickened cIMT subgroup. Despite the small sample size, this finding preliminarily suggests that insulin therapy in our cohort may serve as a marker of more pronounced diabetic disease and subclinical vascular structural changes. This aligns with clinical understanding that insulin initiation often reflects longer disease duration and poorer glycemic control, which are established factors for heightened vascular risk (40). In addition, antiplatelet therapy showed a significant association with cIMT in univariate analysis but was not retained in the multivariable model. Antiplatelet agents are routinely prescribed to reduce thrombotic risk, particularly in patients with established cardiovascular disease or high-risk profiles, their anticipated effect would typically be protective against progression or, alternatively, serve as an indicator of pre-existing elevated cardiovascular risk (41). This discrepancy may be partly explained by the very small number of participants receiving antiplatelet therapy in this study (*n* = 4), which limits the statistical power to detect an independent association (42).

By specifically focusing on increased cIMT in the absence of plaque or significant stenosis, this study characterizes the subclinical vascular profile unique to early-stage T2DM. Consequently, our findings should be interpreted within this specific context and may not be directly generalizable to patients with established atherosclerotic plaques or advanced obstructive disease. However, this focused design allows for a clearer isolation of early biomechanical alterations, which may offer clinical utility for early risk stratification and the implementation of timely primary prevention strategies before irreversible structural damage occurs.

## Study limitations

Several limitations of this study should be noted. First, this was a cross-sectional study, which precludes the determination of a causal relationship between T2DM and carotid biomechanical changes; thus, our findings should be interpreted as associations rather than causal inferences. Second, this was a single-center study with a relatively small sample size (*n =* 92), which may limit the generalizability of the results and the statistical power to detect subtle variations. As an exploratory analysis, these findings are preliminary and require validation in larger, multi-center cohorts. Third, the inability to calculate the Homeostatic Model Assessment of Insulin Resistance (HOMA-IR) due to the lack of routine fasting insulin collection represents a limitation, precluding assessment of its independent association with cIMT. Fourth, focusing solely on the left carotid artery and the common carotid artery in accordance with ASE guidelines, the analysis did not include bilateral assessment or detailed segmental evaluation (e.g., ICA/ECA), nor did it incorporate morphological characterization of cIMT. While this approach helps improve measurement consistency, it may not fully capture regional heterogeneity or more complex vascular features described in current methodological standards and guideline recommendations. Finally, despite incorporating detailed medication records (e.g., antidiabetic agents and statins), the cross-sectional design prevented a full assessment of confounding from varying medication dosages or adherence over time. Longitudinal studies with comprehensive pharmacological data are needed to better isolate the independent effects of diabetic progression on vascular health.

## Conclusion

Ultrasound V-Flow imaging and UF-PWV imaging techniques can be employed to assess the biomechanical properties of the blood vessels. These techniques facilitate the identification of key risk factors and vascular wall dysfunction in the early stages of cIMT thickening in patients with T2DM, thereby providing preliminary evidence to support personalized prevention and treatment strategies. Given the cross-sectional design and relatively small sample size, these findings should be interpreted as exploratory and hypothesis-generating.

## Data Availability

The raw data supporting the conclusions of this article will be made available by the authors, without undue reservation.
