# Supplementary material for: Application of multimodal ultrasound in the biomechanical evaluation of carotid intima-media thickness in type 2 diabetes mellitus: a focus on subclinical vascular changes
Source: Front Endocrinol (Lausanne). 2026 Apr 15;17:1758096. doi: 10.3389/fendo.2026.1758096 (PMC13124614; doi:10.3389/fendo.2026.1758096)
Supplement: Supplementary file 1 [file Table1.docx]

Supplementary Table 1. Univariate and multivariate linear regression analysis of factors associated with intima-media thickness (IMT).

| Variables | Univariate regression analysis | | | | Multivariate regression analysis | | | |
| --- | --- | --- | --- | --- | --- | --- | --- | --- |
|  | B | SE | 95% CI | P value | B | SE | 95% CI | P value |
| Age | 0.004 | 0.003 | (-0.002, 0.011) | 0.204 |  |  |  |  |
| BMI | 0.039 | 0.011 | (0.017, 0.061) | 0.001 | 0.021 | 0.010 | (0.001, 0.041) | 0.044 |
| TC | 0.071 | 0.031 | (0.009, 0.113) | 0.026 | 0.051 | 0.027 | (-0.002, 0.104) | 0.057 |
| TG | 0.020 | 0.041 | (-0.062, 0.101) | 0.633 |  |  |  |  |
| HDL | -0.041 | 0.077 | (-0.194, 0.112) | 0.595 |  |  |  |  |
| LDL | 0.034 | 0..031 | (-0.028, 0.095) | 0.282 |  |  |  |  |
| HbA1c | 0.009 | 0.006 | (-0.002, 0.021) | 0.106 | 0.000 | 0.005 | (-0.010, 0.010) | 0.996 |
| WSSmean | -0.065 | 0.019 | (-0.104, -0.027) | 0.001 | -0.044 | 0.018 | (-0.079, -0.009) | 0.015 |
| PWV-ES | 0.032 | 0.006 | (0.019, 0.045) | < 0.001 | 0.023 | 0.007 | (0.010, 0.036) | 0.001 |
| PWV-BS | 0.023 | 0.010 | (0.003， 0.044) | 0.024 | 0.014 | 0.009 | (-0.005， 0.033) | 0.151 |
| EDV | -0.553 | 0.324 | (-1.196, 0.090) | 0.091 |  |  |  |  |
| PSV | -0.102 | 0.099 | (-0.298, 0.094) | 0.305 |  |  |  |  |
| PI | 0.040 | 0.064 | (-0.087, 0.168) | 0.533 |  |  |  |  |
| RI | -0.220 | 0.296 | (-0.808, 0.369) | 0.460 |  |  |  |  |

Note: Variables with P < 0.05 in univariate regression analysis were included in the multivariate linear regression model. Multivariate results are adjusted for all included variables.
